# Supplementary material for: Selective cell death in HIV-1-infected cells by DDX3 inhibitors leads to depletion of the inducible reservoir
Source: Nat Commun. 2021 Apr 30;12:2475. doi: 10.1038/s41467-021-22608-z (PMC8087668; doi:10.1038/s41467-021-22608-z)
Supplement: Supplementary file 3 — Reporting Summary [file 41467_2021_22608_MOESM3_ESM.pdf]

nature research

Corresponding author(s): Tokameh Mahmoudi

Last updated by author(s): Mar 2, 2021

Reporting Summary

Nature Research wishes to improve the reproducibility of the work that we publish. This form provides structure for consistency and transparency in reporting. For further information on Nature Research policies, see our Editorial Policies and the Editorial Policy Checklist.

Statistics

For all statistical analyses, confirm that the following items are present in the figure legend, table legend, main text, or Methods section.

n/a	Confirmed	
☐	☒	The exact sample size (*n*) for each experimental group/condition, given as a discrete number and unit of measurement
☐	☒	A statement on whether measurements were taken from distinct samples or whether the same sample was measured repeatedly
☐	☒	The statistical test(s) used AND whether they are one- or two-sided<br>*Only common tests should be described solely by name; describe more complex techniques in the Methods section.*
☒	☐	A description of all covariates tested
☐	☒	A description of any assumptions or corrections, such as tests of normality and adjustment for multiple comparisons
☐	☒	A full description of the statistical parameters including central tendency (e.g. means) or other basic estimates (e.g. regression coefficient) AND variation (e.g. standard deviation) or associated estimates of uncertainty (e.g. confidence intervals)
☐	☒	For null hypothesis testing, the test statistic (e.g. *F*, *t*, *r*) with confidence intervals, effect sizes, degrees of freedom and *P* value noted<br>*Give P values as exact values whenever suitable.*
☒	☐	For Bayesian analysis, information on the choice of priors and Markov chain Monte Carlo settings
☒	☐	For hierarchical and complex designs, identification of the appropriate level for tests and full reporting of outcomes
☒	☐	Estimates of effect sizes (e.g. Cohen's *d*, Pearson's *r*), indicating how they were calculated

Our web collection on statistics for biologists contains articles on many of the points above.

Software and code

Policy information about availability of computer code

Data collection	For RT-qPCR, experiments were performed on CFX Connect Real-Time PCR Detection System thermocycler (BioRad) using CFX Manager version 3.0 (BioRad). For microscopy, all phase contrast pictures were acquired using a Leica DMIL microscope and a DFC420C camera. For RNA sequencing, libraries were sequenced according to the Illumina TruSeq v2 protocol on an Illumina HiSeq 2500 sequencer. For Luciferase readouts, data was acquired using GLOMAX (version1.9.2).
Data analysis	For RT-qPCR, data was analysed on CFX Manager version 3.0 (BioRad). For Flow cytometry, data analysis was performed using the FlowJo V10 software (Treestar). Microscopy images Images were analysed and processed using Fiji / ImageJ (NIH). Molecular docking of RK-33 and FH1321 to pre-unwound DDX3 confirmation was performed using Chimera's AutoDock Vina function 71 and figures were created using PyMol Molecular Graphics System, Version 1.8. 2015 (Schrödinger L). For RNA-sequencing, reads were mapped against the UCSC genome browser GRCh38 reference genome with HiSat2 (version 2.1.0), gene expression was quantified using htseq-count (version 0.11.2), differential expression analysis of the RNA-seq data was performed using edgeR package run under Galaxy (https://bioinf-galaxian.erasmusmc.nl/galaxy/), heat maps were generated using MORPHEUS (https://software.broadinstitute.org/morpheus/index.html), volcano plots. For Western blots, signal intensity and densitometry analyses were conducted using ImageJ (NIH). To determine the frequency of cells expressing tat/rev msRNA by maximum likelihood method using TILDA, ELDA software was used. All graphs were generated and statistical analyses were performed using Prism version 8.3.0 (GraphPad software).

For manuscripts utilizing custom algorithms or software that are central to the research but not yet described in published literature, software must be made available to editors and reviewers. We strongly encourage code deposition in a community repository (e.g. GitHub). See the Nature Research guidelines for submitting code & software for further information.

Data

Policy information about availability of data

All manuscripts must include a data availability statement. This statement should provide the following information, where applicable:

- Accession codes, unique identifiers, or web links for publicly available datasets
- A list of figures that have associated raw data
- A description of any restrictions on data availability

All data needed to evaluate the conclusions in the paper are present in the paper and/or the Supplementary Materials. All RNA-seq data has been deposited to the Gene Expression Omnibus (GEO) database with accession code GSE167553 (https://www.ncbi.nlm.nih.gov/geo/query/acc.cgi?acc=GSE167553). Source data are provided with this paper. Additional data related to this paper may be requested from the authors.

Field-specific reporting

Please select the one below that is the best fit for your research. If you are not sure, read the appropriate sections before making your selection.

[x] Life sciences [ ] Behavioural & social sciences [ ] Ecological, evolutionary & environmental sciences

For a reference copy of the document with all sections, see nature.com/documents/nr-reporting-summary-flat.pdf

Life sciences study design

All studies must disclose on these points even when the disclosure is negative.

Sample size	Experiments done with cell line J-Lats 11.1 were performed at least three times, or as indicated in the figure legends. For studies using primary CD4+ T cells obtained from human donors, sample size was at least six , or as indicated in the figure legends. The sample size was determined based on the minimum requirement to perform statistical tests and the availability of materials.
Data exclusions	No data was excluded in these studies.
Replication	All attempts at replication of this data were successful. All experiments had at least three independent replicates and / or technical replicates as indicated in the figure legends.
Randomization	Samples were independently and randomly analyzed.
Blinding	Samples were blindly analyzed.

Reporting for specific materials, systems and methods

We require information from authors about some types of materials, experimental systems and methods used in many studies. Here, indicate whether each material, system or method listed is relevant to your study. If you are not sure if a list item applies to your research, read the appropriate section before selecting a response.

Materials & experimental systems

n/a	Involved in the study
[ ]	[x] Antibodies
[ ]	[x] Eukaryotic cell lines
[x]	[ ] Palaeontology and archaeology
[x]	[ ] Animals and other organisms
[ ]	[x] Human research participants
[x]	[ ] Clinical data
[x]	[ ] Dual use research of concern

Methods

n/a	Involved in the study
[x]	[ ] ChIP-seq
[ ]	[x] Flow cytometry
[x]	[ ] MRI-based neuroimaging

Antibodies

Antibodies used	anti-AnnexinV-450 (560506, BD Biosciences), anti-CD25-PE (555432, Becton Dickinson), anti-Puromycin antibody (12D10 Sigma Aldrich), anti-GAPDH (ab8245, Abcam), anti-DDX3 (ab128206, Abcam), anti- Phospho-IRF-3 (Ser386) (37829, Cell Signalling Tech), anti- HSP70 (4872, Cell Signalling Tech), anti- IRF-3 (sc33641, Santa Cruz), anti BIRC5 (sc17779, Santa Cruz), anti-tubulin (B7, Santa Cruz), BV510 anti-IL2 (563265, BD Biosciences), PE-Cy7 anti-IFNγ (27-7319-41, eBioscience) and horseradish peroxidase-conjugated secondary antibodies (Mouse 610-103-121 and Rabbit 611-103-122; Rockland Immunochemicals).
Validation	All antibodies were validated by the manufacturers and in-house using relevant positive controls. BD biosciences validates antibodies by testing on a combination of primary cells, cell lines and/or transfectant cell models with relevant controls using multiple immunoassays to ensure biological accuracy. They also perform multiplexing with additional antibodies to interrogate antibody

staining in multiple cell populations. 12D10 antobody from Sigma Aldrich was validated by the manufacturer by Western Blotting in HEK293 cell lysates treated with Puromycin and Cyclohexamide, or with Puromycin only. ab8245 was validated by the manufacturer by Western blot using Jurkat cell lysate. ab8128206 was validated by the manufacturer by Western blot using HeLa cell lysate. 37829 was validated by the manufacturer by Western blot analysis of extracts from A549 cells, untreated (-) or treated with Poly (I:C) (+). 4872 was validated by the manufacturer by Western blot using HeLa, NIH/3T3, C6 and COS cell lysate. sc33641 was validated by the manufacturer by Western blot using HL60 cell lysate. sc17779 was validated by the manufacturer by Western blot analysis of survivin expression in COLO 320DM (A), HL-60 (B), MOLT-4 (C) and IMR-32 (D) whole cell lysates. b7 was validated by the manufacturer by Western blot using HeLA and HEL 92.1.7 cell lysate. sc33641 was validated by the manufacturer by Western blot using HL60 cell lysate. 27-7319-41 was validated by treated normal human peripheral blood cells in the presence of Protein Transport Inhibitors (500X) (Unstimulated, bottom row) or Cell Stimulation Cocktail (plus protein transport inhibitors, 500X) for 5 hours (Stimulated, top row). Cells were fixed and permeabilized with the IC Fixation and Permeabilization Buffer Set and protocol followed by intracellular staining with IFN gamma. HRP antobodies from Rockland were prepared from monospecific antiserum by immunoaffinity chromatography using Mouse or Rabbit IgG coupled to agarose beads followed by solid phase adsorption(s) to remove any unwanted reactivities. Assay by immunoelectrophoresis resulted in a single precipitin arc against anti-Peroxidase, anti-Goat Serum, Mouse or Rabbit IgG and Mouse or Rabbit Serum. No reaction was observed against non Mouse or Rabbit sepcies (Bovine, Chicken, Goat, Guinea Pig, Hamster, Horse, Human, Rat and Sheep Serum Proteins).

Eukaryotic cell lines

Policy information about cell lines

Cell line source(s)	J-Lat cell lines 11.1 was obtained from the Verdin and Greene Laboratories at the Gladstone Institutes, which generated these lines from Jurkat cells. Jurkat and HEK293T cells were obtained from the ATCC.
Authentication	J-Lat 11.1 cells were authenticated by the Verdin and Greene Laboratories at the Gladstone Institutes PCR assays with species-specific primers Jurkat and HEK293T cells were authenticated by the ATCC by karyotype analysis.
Mycoplasma contamination	All cell lines were tested negative for mycoplasma testing.
Commonly misidentified lines (See ICLAC register)	No commonly misidentified cell lines were used in this study.

Human research participants

Policy information about studies involving human research participants

Population characteristics	HIV-1 infected donors used in this study were older than 18 years, cART-treated for at least 1 year, with viral loads below 50 copies/ml for more than 2 months. The population characteristics of human research participants are described in Table 3 of the main text.
Recruitment	HIV-1 infected donors were recruited based on voluntary enrollment to participate in the study and provided informed, signed consent. There are no self-selection or other biases that can influence results.
Ethics oversight	The study protocol was approved by the Erasmus Medical Centre Medical Ethics Committee (MEC-2012–583).

Note that full information on the approval of the study protocol must also be provided in the manuscript.

Flow Cytometry

Plots

Confirm that:

- [x] The axis labels state the marker and fluorochrome used (e.g. CD4-FITC).
- [x] The axis scales are clearly visible. Include numbers along axes only for bottom left plot of group (a 'group' is an analysis of identical markers).
- [x] All plots are contour plots with outliers or pseudocolor plots.
- [x] A numerical value for number of cells or percentage (with statistics) is provided.

Methodology

Sample preparation	For FISH-Flow: Primary CD4+ T cells from healthy donors or from HIV-1 infected donors were collected, fixed, permeabilised and subjected to the PrimeFlow RNA assay (Thermo Fisher Scientific) following the manufacturer's instructions. Briefly, cells were first stained in Fixable Viability dye 780 (Thermo Fisher Scientific) for 20 minutes at room temperature (1:1000 in dPBS) followed by either AnnexinV-450 (BD Biosciences) or with 2μM CellEvent™ Caspase-3/7 Green Detection Reagent (Thermo Fisher Scientific) for 30 min at room temperature (1:150 in AnnexinV binding buffer-Biolegend). For both CD4+ T cells and J-Lat 11.1 cells, mRNA was labelled with a set of 40 probe pairs against the GagPol region of the vRNA (catalogue number GagPol HIV-1 VF10-10884, Thermo Fisher Scientific) diluted 1:5 in diluent provided in the kit and hybridized to the target mRNA for 2 hours at 40°C. Positive control probes against the house-keeping gene RPL13A (VA1-13100, Thermo Fisher Scientific) were used to determine assay efficiency. Samples were washed to remove excess probes and stored overnight in the presence of RNAsin. Signal amplification was then performed by sequential 1.5 hour, 40°C incubations with the pre-amplification and amplification mix. Amplified mRNA was labelled with fluorescently-tagged probes for 1 hour at 40°C.

	To determine viability and cell activation following DDX3 inhibition, cells were collected and stained with Fixable Viability Dye eFluor® 780 (Thermo Fisher Scientific) and anti-CD25-PE (Becton Dickinson). Mock-treated and cells treated with PMA-Ionomycin were used as negative and positive controls, respectively. Cells were stained for 30 min at 4 °C, washed with PBS, and resuspended for flow cytometric analysis.<br><br>To analyze T cell proliferation capacity, 1 million CD8+ or CD4+ T cells were stained with 0,07 uM CellTrace Far Red Cell Proliferation dye (Thermo Fisher Scientific) following manufacturer's instructions. Cells were then cultivated for 72 hours with either unstimulated or stimulated conditions in the presence of the DDX3 inhibitors, and analyzed by flow cytometry. Stimulation of T cells was performed using Anti-CD3/CD28 coated Dynabeads (Thermo Fisher Scientific) following manufacturer's protocol.<br><br>To analyze T cell functionality by means of cytokine expression 1 million CD8+ or CD4+ T cells were left untreated or treated with the LRA for 18 hours. Cells were then left unstimulated or stimulated with 50 ng/mL PMA and 1uM Ionomycin for 7 hours in the presence of a protein transport inhibitor (BD GolgiPlugTM, BD Biosciences). To stain for intracellular cytokines cells were washed with PBS supplemented with 3% FBS followed by a fixation and permeabilisation step with FIX & PERM Kit (Invitrogen) following manufacturer's protocol and incubated with 1:25 BV510 anti-IL2 (563265, BD Biosciences) and PE-Cy7 anti-IFNγ (27-7319-41, eBioscience) in permeabilisation buffer for 45 minutes at 4C. Stained cells were washed with PBS supplemented with 3% FBS and analyzed by flow cytometry.
Instrument	Samples were acquired on a BD LSR Fortessa Analyzer.
Software	Data analysis was performed using the FlowJo V10 software (Treestar).
Cell population abundance	For cell line experiments, at least 10,000 cells were counted per condition as indicated in the figure legends. For primary cell experiments from healthy donors, at least 50,000 cells were counted per condition as indicated in the figure legends. For primary CD4+ T cell primary CD4+ T cell experiments from HIV-1-infected donors, at least 100,000 cells were counted per condition as indicated in the figure legends. Purity of CD4+ T cells were routinely tested post extraction from PBMCs by flow cytometry using anti-CD4 Pacific Blue antibody.
Gating strategy	Samples were acquired on a BD LSR Fortessa Analyzer and gates were set using the unstimulated J-Lat 11.1 control sample or uninfected CD4+ T cells For FISH-Flow the gating strategy of the experiments depicted in Figure 3, 4 and 5 are presented in Supplementary Figure 4. For cell line experiments, the gating strategy is FSC-A/SSC-A for cells, FSC-A/FSC-H for single cells, FSC-A/450 for viable cells and FSC-A/530 for GFP+. For all other experiments with primary CD4+ T cells, the gating strategy is FSC-A/SSC-A for cells, FSC-W/FSC-H for single cells, SSC-W/SSC-H for single cells (double), and then gated separately for the different markers (CD25, CD69, IL2 or IFNγ).

☒ Tick this box to confirm that a figure exemplifying the gating strategy is provided in the Supplementary Information.
